# Predictive value of readiness, importance, and confidence in ability to change drinking and smoking

**DOI:** 10.1186/1940-0640-7-S1-A28

**Published:** 2012-10-09

**Authors:** Nicolas Bertholet, Jacques Gaume, Mohamed Faouzi, Jean-Bernard Daeppen, Gerhard Gmel

**Affiliations:** 1 Center for Alcohol Treatment, Department of Medicine and Public Health, Lausanne University, Lausanne, Switzerland

## 

Visual analog scales (VASs) are used to assess readiness to change constructs, which are considered critical for change. We studied whether three constructs—readiness to change, importance of changing, and confidence in ability to change—predict risk status six months later in 20-year-old men with either or both of two behaviors: risky drinking and smoking. Five hundred and seventy-seven participants in a randomized trial of brief intervention (BI) were assessed at baseline and six months on alcohol and tobacco consumption, with three 1-10 VASs (readiness, importance, confidence) for each behavior. We used one regression model for each behavior and construct. Models controlled for receipt of BI and used the VAS lowest level (1-4) as the reference group (versus medium [5-7] and high [8-10] levels). Among the 475 risky drinkers, mean (SD) readiness, importance, and confidence in ability to change drinking were 4.0 (3.1), 2.8 (2.2), and 7.2 (3.0). Readiness was not associated with being free of alcohol risk at six months (OR 1.3 [0.7; 2.2] and 1.4 [0.8; 2.6] for medium and high readiness). High importance and confidence were associated with being risk free (OR 0.9 [0.5; 1.8] and 2.9 [1.2; 7.5] for medium and high importance; 2.1 [1.0; 4.8] and 2.8 [1.5; 5.6] for medium and high confidence). Among the 320 smokers, mean readiness, importance, and confidence to change were 4.6 (2.6), 5.3 (2.6), and 5.9 (2.6). Neither readiness nor importance was associated with being smoking free (OR 2.1 [0.9; 4.7] and 2.1 [0.8; 5.8] for medium and high readiness; 1.4 [0.6; 3.4] and 2.1 [0.8; 5.4] for medium and high importance). High confidence was associated with being smoking free (OR 2.2 [0.8; 6.6] and 3.4 [1.2; 9.8] for medium and high confidence). For drinking and smoking, high confidence in ability to change was associated, with similar magnitude, with a favorable outcome. This points to the value of confidence as a predictor of successful change.

